# Cutaneous varicella zoster virus infection: an assocation with ibuprofen?

**DOI:** 10.31744/einstein_journal/2019AI4809

**Published:** 2019-10-03

**Authors:** Joana Jorge Antunes, Susana Silva Dias, Ricardo Miguel Patrício de Carvalho Monteiro, Ana Mafalda Martins

**Affiliations:** 1 Hospital de Cascais Dr. José de Almeida Alcabideche Portugal Hospital de Cascais Dr. José de Almeida, Alcabideche, Portugal.

Varicella is a viral and usually benign disease, which commonly affects children. This disease main complication is bacterial superinfection of the skin.^1^ Ibuprofen administration, although not contraindicated, seems to increase risk of severe skin complications.^2 - 5^

**Figure 1:**
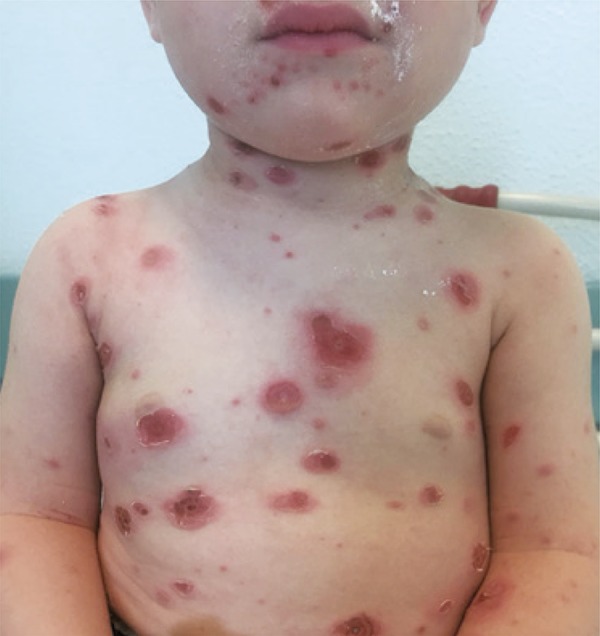
Varicella zoster lesions on chest

**Figure 2:**
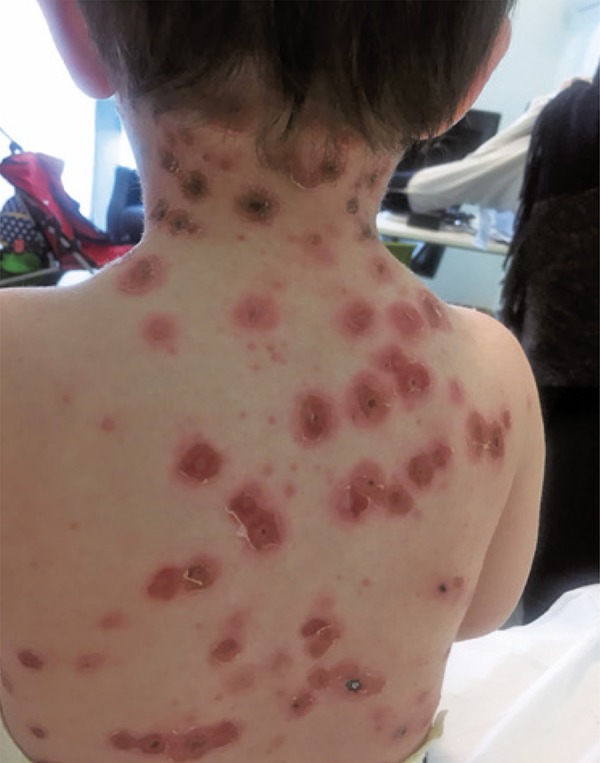
Varicella zoster lesions on the back

We report a case of a 21-month-old child with no family or personal relevant medical history who developed varicella with high fever since the second day of the disease. Paracetamol 15mg/kg every 8 hours and ibuprofen 7mg/kg every 8 hours were administered to the child after the onset of fever. Six days after the disease onset, because of the worsening of skin lesions, pain on mobilization and touch, the patient was taken to emergency service. Upon admission the patient presented exuberant exanthema all over the tegument, including scalp and mucosae, and lesions in different evolution stages. There were multiple hardened ulcer base lesions on the chest and back, surrounded by erythematous halo – two of them very painful on touch ( Figures 1 and 2 ). The patient was hospitalized and the ecography of soft parts did not show depth penetration. After 14-days therapy with flucloxacillin 150mg/kg/day, and 10-days therapy with clindamycin 25mg/kg/day the patient’s clinical picture improved progressively with reduction of pain and amelioration of inflammatory skin lesions.

Although benign, varicella-associated complications are frequent. Studies have reported potential increase in risks and severe skin associated complications with the use of non-steroidal anti-inflammatory (NSAI),^2 , 3^ however, without proved relationship with necrotizing fasciitis.^1 , 6^ Exposure to ibuprofen compromises the leukocytosis function, promotes an increase of inflammatory cytokine production,^1 , 2 , 5^ and creates a convenient environment for bacterial growth. Some authors believe that ibuprofen administration may hidden symptoms and may lead to a delay in diagnosis.^4^

The health professional is responsible for providing counseling for parents about the use of NSAI, which sometimes are administered without formal medical advice. Further studies are warranted to conclude the safety of these drugs during varicella infection. Currently, the use of NSAI during varicella infection must be avoided.^7^
