# Supplementary material for: Cutaneous varicella zoster virus infection: an assocation with ibuprofen?
Source: Einstein (Sao Paulo). 2019 Oct 3;17(4):eAI4809. doi: 10.31744/einstein_journal/2019AI4809 (PMC6777742; doi:10.31744/einstein_journal/2019AI4809)
Supplement: Translation

# Translation: Sobreinfecção cutânea de varicela: uma associação com ibuprofeno?

**Authors:** Joana Jorge Antunes, Susana Silva Dias, Ricardo Miguel Patrício de Carvalho Monteiro, Ana Mafalda Martins

**Affiliations:** 1 Portugal Hospital de Cascais Dr. José de Almeida, Alcabideche, Portugal.

A varicela é uma doença viral, comum em idade pediátrica e habitualmente benigna, tendo como principal complicação a sobreinfecção bacteriana da pele.^1^ A administração de ibuprofeno, apesar de não ser interdita, parece aumentar o risco de complicações cutâneas graves.^2 - 5^

Apresenta-se o caso de uma criança com 21 meses de idade, sem antecedentes pessoais ou familiares relevantes, que iniciou quadro de varicela associado a febre alta desde o segundo dia de doença, tendo sido medicada com paracetamol 15mg/kg a cada 8 horas e ibuprofeno 7mg/kg também a cada 8 horas. No sexto dia da doença, por agravamento das lesões cutâneas, e com dor à mobilização e ao toque, recorreu ao serviço de urgência. À observação, destacava-se exantema exuberante por todo o tegumento, incluindo couro cabeludo e mucosas, com lesões em vários estádios de evolução. No tronco e dorso, existiam múltiplas lesões endurecidas, de base ulcerada, circundadas por halo eritematoso − duas delas significativamente dolorosas ao toque ( Figuras 1 e 2 ). Ficou internado, tendo realizado ecografia de partes moles, que não demonstrou atingimento profundo. Cumpriu 14 dias de flucloxacilina, na dose de 150mg/kg/dia, e 10 dias de clindamicina, na dose de 25mg/kg/dia, com melhoria progressiva das queixas álgicas e sinais inflamatórios cutâneos.

**Figura 1:** Lesões de varicela no tronco

**Figura 2:** Lesões de varicela no dorso

Apesar de sua benignidade, as complicações da varicela são frequentes. Estudos têm demonstrado potencial aumento do risco de complicações cutâneas graves em associação ao uso de anti-inflamatórios não esteroides (AINEs),^2 , 3^ sem, no entanto, relação comprovada com fasceíte necrotizante.^1 , 6^ Pensa-se que a exposição ao ibuprofeno compromete a função leucocitária e promove aumento de produção de citocinas inflamatórias,^1 , 2 , 5^ criando ambiente propício para crescimento bacteriano. Alguns autores defendem ainda que sua administração, ao mascarar os sintomas, possa levar a um atraso diagnóstico.^4^ Cabe aos profissionais de saúde providenciar aconselhamento aos pais sobre a utilização de AINEs, muitas vezes administrados sem indicação médica formal. Estes fármacos devem ser evitados durante o período de varicela, até o surgimento de estudos que comprovem sua segurança.^7^
